# The evaluation crisis in artificial intelligence‐driven computational biology: Pitfalls and solutions

**DOI:** 10.1002/qub2.70058

**Published:** 2026-09-23

**Authors:** Tsung Fei Khang, Joshua W. K. Ho, Lin Hou, Jiangning Song, Limsoon Wong

**Affiliations:** ^1^ Institute of Mathematical Sciences, Faculty of Science Universiti Malaya Kuala Lumpur Malaysia; ^2^ Universiti Malaya Centre for Data Analytics Universiti Malaya Kuala Lumpur Malaysia; ^3^ School of Biomedical Sciences, Li Ka Shing Faculty of Medicine The University of Hong Kong Hong Kong China; ^4^ School of Biomedical Engineering The University of Hong Kong Hong Kong China; ^5^ Centre for Health AI Research and Translation (CHART), Li Ka Shing Faculty of Medicine The University of Hong Kong Hong Kong China; ^6^ Department of Statistics and Data Science Tsinghua University Beijing China; ^7^ Biomedicine Discovery Institute and Department of Biochemistry and Molecular Biology Monash University Melbourne Victoria Australia; ^8^ School of Computing National University of Singapore Republic of Singapore

**Keywords:** artificial intelligence, biological validity, computational biology, generalization, model evaluation

## Abstract

Artificial intelligence (AI) is rapidly transforming computational biology. However, closer scrutiny reveals that many reported results do not hold under rigorous evaluation. This paper identifies prevalent, recurring issues in the field, including narrow datasets, hidden confounders that make supposedly independent test data effectively indistinguishable from the training data (the doppelgänger effect), misleading performance metrics, and over‐reliance on overly easy test cases. We argue that meaningful assessment must go beyond metric‐centric model evaluation to encompass biological realism, data diversity, confounding control, and interpretability. Crucially, AI studies in computational biology should deliver not only predictive performance but also genuine biological insight and transferable methodological understanding. To address these systemic limitations, we propose a unifying framework, VALID, comprising the following five guiding principles for rigorous AI in biology: Veracious biological grounding and novelty, artifact control and methodological rigor, legitimate generalization, inspectable preprocessing, and deployment‐aware performance. Together, these principles serve as a diagnostic lens for structuring our discussion throughout this paper. We further illustrate common pitfalls through representative examples and provide actionable guidance for authors, reviewers, and editors aimed at improving the rigor and scientific value of AI‐driven computational biology.

## 1 COMPUTATIONAL BIOLOGY AS A PROVING GROUND FOR AI

Computational biology has become a central arena for artificial intelligence (AI), with a substantial share of contemporary research now relying on AI‐based methods. From biomarker discovery to protein function prediction, AI is frequently framed as a key enabler for uncovering biological mechanisms. Yet a more sobering pattern has emerged: many reported results do not survive careful re‐examination. The underlying concern is not simply model accuracy, but the reliability of the evaluation frameworks themselves.

Meaningful assessment requires more than numerical performance metrics. It must incorporate realistic experimental conditions, heterogeneous and diverse populations, careful control of confounding variables, and genuine biological interpretability. Equally important is alignment with real‐world constraints and deployment contexts. Too often, problems are formulated primarily as publication vehicles, with limited consideration of how predictions will inform biological decisions or integrate into experimental or clinical workflows. This misalignment often reflects insufficient biological grounding and limited engagement with domain stakeholders. Without adequate molecular biology literacy, computational researchers risk missing essential assumptions, overlooking failure modes, and designing systems that are difficult to interpret or translate. Even technically sophisticated models may therefore fail at the point of use.

To address these recurring limitations, we introduce VALID, comprising five guiding principles for rigorous AI in biology (Figure 1):

1. Veracious biological grounding & novelty: Ensuring that model development is anchored in biological relevance and yields testable scientific insight.
2. Artifact control & methodological rigor: Addressing statistical validity, leakage, and hidden confounders that can inflate apparent performance.
3. Legitimate generalization: Emphasizing robust testing under challenging and realistic conditions, beyond curated benchmarks.
4. Inspectable preprocessing: Making explicit how data processing steps may introduce bias or distort biological signals.
5. Deployment‐aware performance: Evaluating models in light of real‐world constraints, use cases, and translational utility.

**FIGURE 1:**
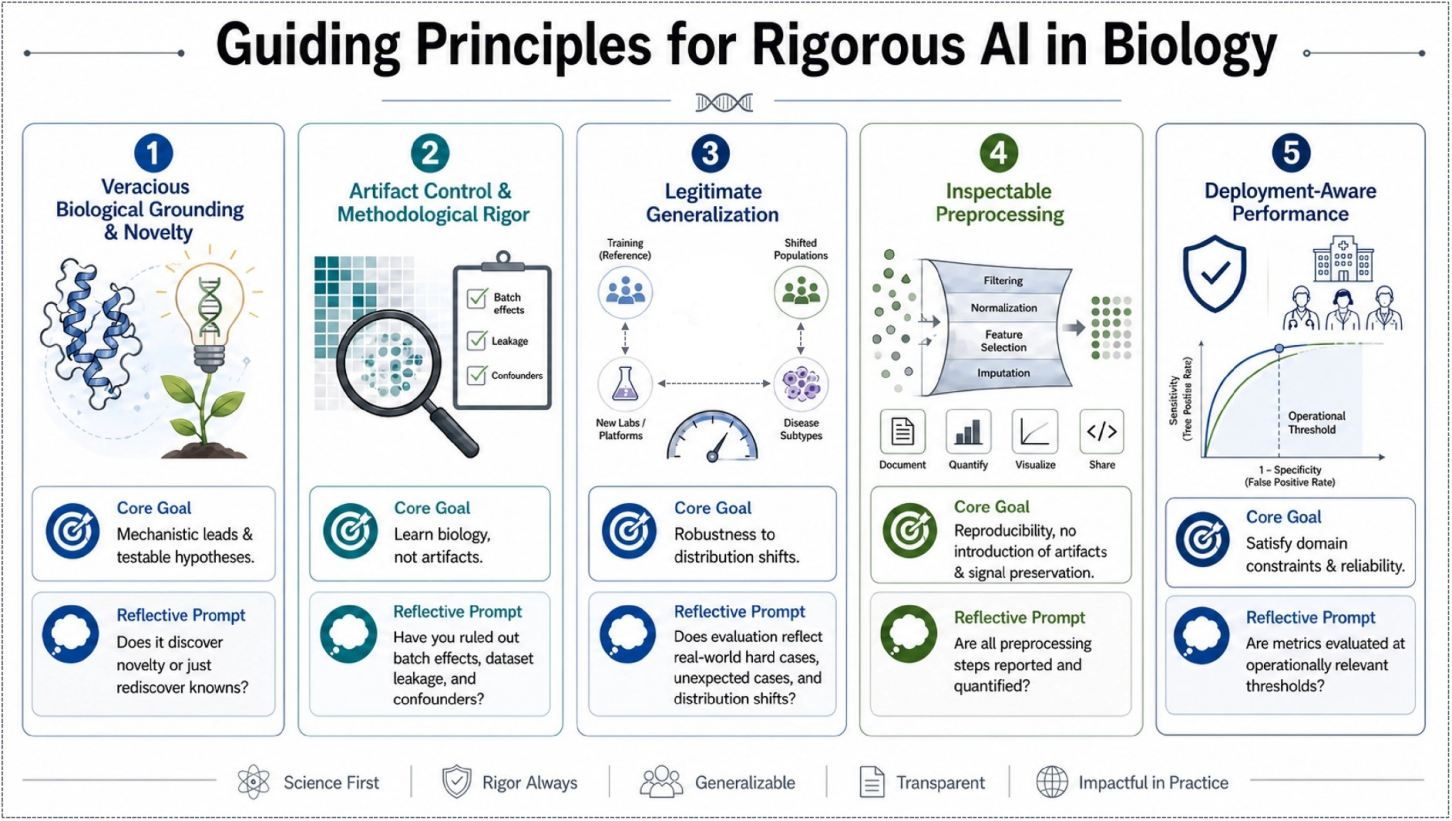
The five VALID principles for rigorous artificial intelligence in biology. These pillars provide the diagnostic criteria used herein: (1) Veracious biological grounding & novelty; (2) Artifact control & methodological rigor; (3) Legitimate generalization; (4) Inspectable preprocessing; and (5) Deployment‐aware performance. (The image was produced by prompting Manus 1.6 Lite.)

This framework provides a unified lens for diagnosing methodological fragility in AI‐driven biological research. Each principle targets a distinct dimension of robust evaluation. Together, these principles provide the structure for the analysis that follows, turning isolated examples into broader methodological lessons. The aim is to encourage a research culture in which AI for biology is judged not only by predictive performance, but by its ability to produce reliable, interpretable, and biologically meaningful contributions that can withstand real‐world use.

## 2 THE ILLUSION OF SIGNIFICANCE: STATISTICAL SIGNIFICANCE IS NOT BIOLOGICAL VALIDITY

The pursuit of statistical significance often overshadows the more difficult goal of establishing biological validity in AI‐driven computational biology. A canonical example is the work by Venet et al. [1], who showed that many random gene signatures, constructed without any biological rationale, can nonetheless produce highly significant *p*‐values for cancer prognosis. This result carries a clear implication: apparent significance is not evidence of biological meaning. Robust biomarkers are distinguished not by performance on a single dataset, but by reproducible performance across multiple independent datasets and conditions [2]. Despite this, much AI‐driven biomedical research continues to rely on single‐dataset evaluations, with peer review often accepting such limited validation. Although reproducing a state‐of‐the‐art deep learning model from scratch is non‐trivial and requires resources that are unavailable to many researchers, small‐scale deep learning applications can often be reproduced with modest computing resources [3]. This distinction is important: although full reproduction of large models may be unrealistic, we should still expect some level of independent verification. Without broad external replication, claims of discovery can easily reflect dataset‐specific artifacts rather than true biological signals [4]. We suggest that the same concern applies to statistical interpretation more generally. Persistent frustration with uncritical dichotomous interpretation of *p*‐values in the literature led to an open letter endorsed by 854 scientists from 52 countries, calling on researchers to move away from statistical significance toward interpretation of point estimates and uncertainty intervals in the context of the scientific problem under study [5].

A related and equally pervasive weakness is the lack of mechanistic or conceptual justification for why a model should work in the first place. Authors often do not specify the biological signals their models are intended to capture, how architectural choices encode inductive biases aligned with biology, or why these design decisions should improve robustness or transferability. As a result, essential sanity checks are frequently absent. These include the use of negative controls, systematic input perturbations to test invariances, and validation that learned representations align with known biological structure. In their absence, even models with high area under the receiver operating characteristic curve (AUROC) or low *p*‐values remain statistically impressive but scientifically unconvincing.

A further distinction that is often blurred is that between explainability and justifiability. Explainability refers to how a model produces a prediction, whereas justifiability concerns whether that prediction is scientifically or clinically defensible [6]. The latter is essential for accountability, safety, and eventual regulatory acceptance. Post hoc explanation methods such as SHAP [7], LIME [8], and Grad‐CAM [9] can provide useful diagnostic insight, but they are inherently context‐dependent and may not reflect stable biological mechanisms. Where suitable, running multiple post hoc explanations using diverse sets of hyperparameters and checking the results for consensus may lead to more robust interpretations [10].

In many cases, domain‐informed ablation studies grounded in explicit biological hypotheses provide more robust and interpretable evidence. For a model to be credible, its outputs must be consistent with established biology, and any deviations should be clearly framed as testable hypotheses, with explicit justification for their scientific relevance rather than post hoc rationalization.

To move beyond the illusion of significance, the field must raise the standard of validation. This includes replication across datasets, conditions, and laboratories; explicit articulation of biological plausibility; and routine incorporation of sanity checks and feature‐level analyses. Early and sustained engagement with domain experts is essential to ensure that both problem formulation and evaluation criteria reflect real biological and clinical needs. Finally, interpretability tools should be used with care, with preference given to hypothesis‐driven, biologically grounded analyses rather than purely post hoc explanations.

## 3 HIDDEN CONFOUNDERS AND THE DOPPELGÄNGER PROBLEM: MODELS LEARN SHORTCUTS

AI models, in their pursuit of optimal predictive performance, often learn shortcuts, which are decision rules that perform well on standard benchmarks but fail to transfer to more challenging real‐world scenarios because they do not learn the underlying biological mechanisms [11]. This failure mode arises when models exploit spurious signals in the data—ranging from confounding factors and dataset artifacts to data leakage—leading to inflated performance metrics that do not reflect genuine biological understanding. An instance of this phenomenon, termed the doppelgänger problem, occurs when test instances are near‐duplicates or near‐identical counterparts of training instances, resulting in data leakage with concomitant artificially inflated performance. A well‐known illustration is the prediction of lung cancer mutations directly from histopathology images [12]. Although the model was initially reported as highly promising, subsequent analyses suggested that it may have exploited confounding factors or even duplicated samples across training and test splits [13]. Therefore, data leakage is not merely a technical flaw in model evaluation; it also harms reproducibility, since models that rely on accidental regularities for strong performance cannot be expected to retain it when challenged with genuinely independent data [14]. More broadly, shortcut learning may arise from batch effects, staining or imaging artifacts, site‐specific signatures, or dataset redundancies—all of which can cause models to encode spurious correlations rather than robust biological signals [15].

The continued publication of implausibly strong performance on narrow or weakly controlled datasets, often without serious interrogation of confounding, effectively normalizes fragile scientific practice. This leads to a recurring cycle in which apparent “breakthroughs” fail to generalize beyond the original dataset or experiment setting. Although methods such as uncertainty‐aware prediction (e.g., Hie et al. [16]) and explicit abstention mechanisms cannot fully eliminate shortcut learning or data leakage, they can reduce downstream harm by preventing overconfident predictions under distribution shift and by flagging atypical inputs for expert review. Complementary strategies, however, are also emerging as a response to such shifts after deployment: Subasri et al. [17] recently demonstrated that data shifts that degrade the performance of a clinical AI system used to predict in‐hospital mortality can be mitigated using transfer learning and drift‐triggered continual learning strategies.

Mitigating the doppelgänger problem requires systematic and explicit checks for confounding and leakage as a default requirement rather than an afterthought. Models should be evaluated across multiple independent datasets, ideally drawn from different laboratories, platforms, or collection protocols, to test robustness against dataset‐specific artifacts. Studies should also include explicit discussion of potential shortcuts and failure modes as part of standard reporting. In addition, incorporating uncertainty‐aware outputs and enabling “unknown” or abstain options can improve transparency, ensuring that models communicate their limitations rather than masking them behind confident but unreliable predictions.

## 4 WHEN ACCURACY MISLEADS: METRICS MUST REFLECT DEPLOYMENT

The reliance on accuracy as a primary metric in computational biology is often misleading, largely because test sets rarely reflect real‐world deployment conditions. Differences in disease prevalence, case mix (including age, sex, ancestry, and disease stage), site‐specific protocols, and instrumentation can all make headline performance figures deceptive. As a result, a model that performs well on a convenience sample may fail when deployed in practice.

To ensure that reported metrics meaningfully reflect deployment utility, authors must go beyond test‐set performance and report expected performance under real‐world conditions [18, 19]. This requires clearly describing how the test set differs from the intended deployment environment, providing subgroup‐stratified performance results, and translating metrics to the target setting using approaches such as reweighting or recalibration. Under prevalence shift, sensitivity and specificity may remain stable, but positive and negative predictive values (PPV/NPV) must be recomputed for the target prevalence. Under covariate shift, subgroup‐level estimates of sensitivity and specificity should be reweighted to match the deployment distribution. In all cases, both raw and deployment‐adjusted metrics should be reported explicitly, together with the assumptions required for extrapolation and, where possible, validation on external cohorts.

Performance evaluation must also focus on operationally meaningful regions of a model’s output space rather than global averages alone. A high AUROC can still coexist with clinically unacceptable sensitivity or specificity at relevant decision thresholds. In line with Simon’s principle [20] of satisficing, models should be assessed at thresholds that satisfy domain‐specific constraints rather than optimized aggregate metrics. In addition, metrics such as PPV and accuracy are strongly prevalence‐dependent; when reported, they must be interpreted in context, ideally using frequency‐based formulations that improve interpretability and reduce reasoning errors [21]. Metric selection should therefore be guided not only by statistical properties but also by their relevance to real decision‐making.

Ultimately, meaningful evaluation requires explicit alignment between test conditions and deployment reality. This includes documenting test–deployment differences, reporting subgroup‐stratified results, and presenting both raw and deployment‐adjusted estimates with uncertainty and clearly stated assumptions. It also requires evaluating models at operationally relevant thresholds that satisfy domain constraints, alongside external validation on independent sites or cohorts whenever feasible.

## 5 EASY QUESTIONS, HARD QUESTIONS, AND SURPRISES: GENERALIZATION IS REVEALED BY DIFFICULT AND UNEXPECTED CASES

AI evaluations in biology often overemphasize “easy questions,” focusing on familiar, well‐represented, or near‐duplicate cases. This introduces a subtle but important bias: performance appears stronger than it truly is, reflecting an overreliance on familiar patterns and a neglect of harder, less typical instances [22]. Yet genuine generalization is only revealed when models are tested on difficult and unexpected cases. Robust evaluation therefore requires test sets that intentionally include challenging instances rather than convenience samples.

The DeepFam predictor [23] provides a clear cautionary example. Although it performed well on proteins closely resembling those seen during training, its performance collapsed in two critical regimes [24]. First, for “twilight zone” proteins—low‐similarity sequences within known functional families—the model struggled significantly. Second, for proteins belonging to functions entirely absent from the training data, DeepFam produced confident yet incorrect predictions. In contrast, proteins outside the twilight zone are often readily classified even by simpler baseline methods, underscoring how apparent gains can be concentrated in easier regions of the problem space.

These failure modes highlight the importance of uncertainty‐aware and open‐set prediction frameworks. Rather than forcing every input into a known training category, models should be able to signal when a query lies outside their reliable domain. Approaches that quantify predictive uncertainty, detect out‐of‐distribution sequences, or allow “none of the above” responses can help expose these limits. Although such methods do not eliminate errors, they make model competence boundaries explicit, reducing the risk of overconfident but incorrect biological conclusions.

A key insight is that difficulty itself should be treated as a first‐class axis of evaluation. Sample difficulty can, for example, be quantified using measures such as the entropy of class labels among *k*‐nearest neighbors in feature space. Performance often degrades sharply on such “hard” samples even when aggregate metrics remain strong—an effect reminiscent of Simpson’s [25] paradox, where overall trends mask important subgroup failures. Table 1 shows a hypothetical example in which prediction accuracy is stratified by sample hardness. Method B achieves higher accuracies than Method A for both easy samples and hard samples, with accuracies of 0.99 and 0.60 compared with 0.95 and 0.50, respectively. However, if Method A is evaluated on a test set dominated by easy samples, its aggregate accuracy reaches 0.905, substantially exceeding Method B’s aggregate accuracy of 0.639. This apparent superiority is misleading, however, because Method B is evaluated on a test set containing a much larger proportion of hard samples. To uncover these hidden weaknesses, evaluations should therefore stratify test data by difficulty or similarity, such as sequence‐similarity bins in protein tasks, to map the true boundaries of generalization.

**TABLE 1:** Expected accuracy of two different models evaluated on two test sets with different compositions of test sample difficulty.

Sample hardness	Method A	Method B
Easy	855/900	99/100
Hard	50/100	540/900
Total	905/1000	639/1000

Progress in the field depends on explicitly targeting these hard cases where current methods fail. These cases are not merely edge cases; they often provide the most informative signal about both model limitations and underlying biology. Reporting should therefore emphasize performance on challenging instances rather than relying primarily on aggregated results from convenient splits. This shift is essential for developing AI systems that are not only accurate on average, but reliable under realistic conditions.

To support this, evaluation design should move away from convenience splits toward more demanding protocols such as leave‐family‐out, time‐split, or lab‐split evaluations that better reflect deployment conditions. Test sets should deliberately include low‐similarity and out‐of‐class examples, with difficulty explicitly defined and reported. Studies should also report performance stratified by difficulty, alongside careful analysis of failure cases that are systematically examined rather than anecdotal. Finally, attention to Simpson’s paradox through joint reporting of aggregate and subgroup results, together with uncertainty‐aware or open‐set prediction, can further clarify the true limits of model generalization.

## 6 THE MIRAGE OF BATCH CORRECTION: PREPROCESSING CAN DESTROY BIOLOGY

Preprocessing is often treated as a routine and largely benign step in computational biology workflows, yet it can introduce a subtle but serious risk: the appearance of improved signal can come at the cost of erasing real biological structure. In single‐cell analysis, for example, tools such as Seurat [26] are widely used for batch effect correction. However, overly aggressive harmonization can eliminate biologically meaningful variation. Yong et al. [27] provided a striking illustration: in an effort to identify rare premalignant cells—mutant populations that closely resembled their normal counterparts—batch correction merged these rare cells into parental clusters. Although batch differences were successfully reduced, the procedure simultaneously removed the very signal of biological interest, undermining the analytical objective—to borrow the old adage: “the operation was successful, but the patient died!”

This example highlights an often‐underappreciated distinction between representativeness of the target biological population and reliability of observed measurements. Representativeness is frequently implicit and difficult to verify, yet it must be explicitly described through sampling design and coverage assumptions. Reliability, in contrast, should be assessed using domain‐appropriate quality control (QC) procedures, such as FastQC for sequencing data, with exclusion criteria clearly justified. When applied appropriately, domain‐informed QC can improve signal‐to‐noise ratios by reducing technical variability without compromising biological signal.

A further complication arises from the assumptions embedded in batch correction and normalization methods. Many are developed under implicit assumptions of sample homogeneity, such as shared phenotypes across batches. When these methods are applied to heterogeneous datasets or settings with uneven phenotype distributions, their underlying assumptions may be violated. In such cases, correction may need to be class‐specific or otherwise adapted to preserve structured biological variation; otherwise, meaningful biological differences risk being removed alongside technical artifacts.

Missing data introduce an additional layer of difficulty. Most batch correction methods are not designed to operate in the presence of missing values, whereas imputation methods typically ignore batch structure. This creates a fundamental ordering problem: should imputation occur before or after batch correction? Each choice carries risks. Imputing first may propagate batch‐specific artifacts, whereas correcting first may distort underlying biological structure. In practice, careful and often iterative or hybrid strategies are needed to balance these competing effects [28].

Taken together, these issues underscore a broader principle: preprocessing tools are not inherently problematic, but their impact depends critically on whether their assumptions match the data‐generating process. Blind application can silently replace one form of distortion with another, producing results that appear cleaner while being further removed from biological reality.

Avoiding this “mirage of batch correction” requires deliberate and transparent practice. Researchers should begin with a clear account of data quality and sampling scope, including explicit discussion of representativeness and justification of exclusions using appropriate QC. Batch correction and normalization should be selected and applied in alignment with dataset structure, particularly in heterogeneous settings where stratified or class‐aware approaches may be necessary. Biological preservation should be actively verified by comparing results with and without correction and checking for retention of known structures. Missing data handling must be explicitly justified, especially in relation to its ordering with respect to batch correction. Robustness should be assessed through sensitivity analyses, including perturbations or synthetic mixing experiments, to detect overcorrection. Finally, comprehensive documentation of preprocessing decisions and their interactions are essential to ensure reproducibility and enable meaningful critique.

## 7 BEYOND NUMBERS: SCIENTIFIC VALUE COMES FROM INSIGHT

Perhaps the most limiting habit in AI‐driven computational biology is the fixation on predictive metrics alone. A model that is accurate but offers no explanation or mechanistic grounding is, at best, a statistical curiosity rather than a scientific contribution. Although many studies conclude by reporting AUROC or accuracy, they often provide little insight into underlying biological processes. Rarely do they interrogate learned representations for motifs, positional dependencies, structural constraints, or signals of evolutionary conservation. Even fewer translate these representations into experimentally testable hypotheses. Yet scientific value arises from insight, not numbers.

Careful quantitative analysis of biologically meaningful properties, whether molecular, structural, or morphological, can reveal patterns and functional relationships that are not visible through superficial inspection [29]. Such analyses form a bridge between predictive performance and biological understanding. Interpretability methods can then help translate these patterns into concrete hypotheses for experimental validation. However, these tools are not sufficient on their own; their outputs must be triangulated against established domain knowledge and supported by rigorous ablation studies. At the same time, models should report calibrated uncertainty and incorporate out‐of‐distribution detection, with explicit criteria for flagging low‐confidence or atypical cases for expert review or downstream assays. The ability of models to signal uncertainty—whether aleatoric, where the model effectively says, “I cannot decide because the observation itself is noisy or ambiguous” [30], or epistemic, where the model says, “I do not know because this instance lies outside my learned domain” [16, 31, 32]—is not a weakness, but a mechanism that can enable targeted experiments and, in some cases, reveal genuinely novel biological classes.

Beyond immediate experimental utility, computational studies should also generate methodological insight for the field. Strong work should clarify which inductive biases are effective (e.g., structure‐aware or motif‐aware architectures), which features remain stable across cohorts, how preprocessing choices affect signal integrity, how uncertainty should be modeled, and where optimization objectives diverge from scientific goals. Importantly, negative results are equally informative: they expose when models overfit to dataset‐specific shortcuts, when batch correction suppresses rare but meaningful biology, or when calibration breaks under distribution shift.

It is therefore necessary to move beyond the assumption that higher performance metrics imply greater scientific value. The goal is not only prediction, but understanding. Models should be systematically interrogated for biological insight and methodological lessons, explicitly connected to testable hypotheses, stress‐tested on challenging cases, and linked to experimental validation where possible. By prioritizing interpretability, mechanistic reasoning, and methodological learning, computational studies can move beyond black‐box performance reporting toward genuine scientific contribution that informs targeted experiments and advances the field.

To support this shift, researchers should treat metrics as necessary but insufficient, complementing them with interpretability and mechanistic analysis. Ablation and stress testing should be used to expose model limitations, and findings should be translated into explicit, testable hypotheses. Negative results and failure cases should be analyzed rather than discarded, as they often reveal structural weaknesses in models or datasets. Finally, uncertainty‐aware prediction and anomaly detection should be incorporated to avoid overconfident outputs and to surface potentially novel biology.

## 8 FROM PROOF‐OF‐CONCEPT TO PRODUCTION: DEPLOYMENT IS A SCIENTIFIC ISSUE

Academic studies in computational biology often underemphasize the practical constraints that determine whether models remain useful beyond the paper. Real‐world deployment requires more than a trained model: it demands continuous monitoring for distributional drift, recalibration mechanisms, robust and reproducible data pipelines, containerized or otherwise reproducible environments with pinned dependencies, and sustained operational expertise. These requirements typically exceed what is available in standard academic settings. This gap is reinforced by incentives that favor novelty and benchmark gains over robustness and long‐term reliability. Deployment should therefore be treated not as an afterthought, but as a scientific concern tightly linked to model validity and utility. Seneviratne et al. [33] advocated that effective bridging of AI‐driven model development and deployment in healthcare requires actionable and practical model recommendations with appropriate user‐experience design, safety guardrails (e.g., is there risk of external validity failure leading to serious patient consequences), and a cost‐utility assessment (i.e., savings from early intervention vs. wastage from unnecessary investigations).

Thus, bridging this gap requires early engagement with all relevant stakeholders, including end‐users, domain scientists, clinicians, industry partners, and funding agencies, to define realistic deployment contexts from the outset. Although AI model development can be computationally intensive, collaboration with industry can provide access to large‐scale infrastructure for training, evaluation, and production. At the same time, rigorous evaluation does not depend primarily on computers. Even smaller laboratories can meet high standards through careful experiment design, forensic analysis of failure cases, and stratified evaluation strategies grounded in domain expertise rather than computational scale. In this sense, strong evaluation practices level the playing field, enabling meaningful scrutiny of model reliability prior to deployment.

Ultimately, these guidelines empower users to critically assess whether reported performance is scientifically meaningful before adopting a tool. This requires attention to independent and representative evaluation sets, as well as explicit deployment‐aware assessment practices. Without such scrutiny, reported performance can be misleading, and unreliable models may enter use. Adhering to these rigorous standards also ensures that researchers and reviewers avoid releasing unreliable models into the community.

To support successful translation from proof‐of‐concept to production, deployment assumptions must be stated explicitly and potential data shifts anticipated. Studies should describe monitoring and update strategies such as drift detection, recalibration, and scheduled revalidation. Reproducibility should be ensured through reporting of random seed sensitivity, run‐to‐run variability, and environmental dependence. Early stakeholder engagement helps align model outputs with real decision‐making workflows, whereas external collaborations may support the infrastructure required for large‐scale deployment. Framing deployment in this way makes it an integral part of scientific validation rather than a downstream engineering concern.

## 9 TOWARD A HIGHER STANDARD FOR PEER REVIEW: INCENTIVES AND REVIEW STANDARDS MUST CHANGE

The recurring failures in AI‐driven biology—ranging from narrow datasets and hidden confounders to misleading metrics, absence of hard cases, overconfidence in closed‐world assumptions, preprocessing‐induced distortions, and weak mechanistic grounding—collectively indicate a systemic lowering of scientific standards. If uncorrected, the field risks normalizing a lower evidentiary threshold. Raising the standard therefore requires a cultural shift, grounded in a re‐examination of incentives, evaluation norms, and peer‐review expectations.

As authors, this implies moving beyond superficial optimization of benchmark performance. Studies should prioritize datasets that reflect true biological diversity, ensure that test sets are genuinely independent and representative [34], and explicitly connect predictive outputs to biological mechanisms or hypotheses. As reviewers and editors, emphasis must shift toward reproducibility, interpretability, and biological relevance, rather than marginal gains on aggregate metrics. The impact of AI in biology should not be measured by publication volume or leaderboard performance, but by whether results withstand independent scrutiny and contribute meaningfully to biological understanding. Equally important is the distinction between genuine discovery and the re‐packaging of known results under new computational frameworks, the latter often reflecting parameter tuning around pre‐existing expectations rather than new biological insight [34].

To embed these principles into practice, journals must move beyond one‐time peer review and introduce structural mechanisms for ongoing scientific accountability. One promising direction is the creation of a dedicated venue for forensic analysis of failure modes. Instead of focusing solely on novelty or incremental improvements, this track would encourage rigorous re‐evaluations of prior work, explicitly targeting systemic issues such as the doppelgänger problem, metric misinterpretation, or preprocessing‐induced signal loss. Such analyses are often most compelling when supported by transparent synthetic datasets with known ground truth, enabling clear identification of whether models are capturing true biological signals or exploiting artifacts that would otherwise remain hidden in complex real‐world data. To this end, documenting the reporting details of machine learning‐based science such as the REFORMS checklist can be invaluable for helping researchers to clarify their methodology and assisting reviewers in identifying potential issues [35].

The value of this type of forensic re‐evaluation is already evident in several studies cited earlier: Oner et al. on [13] lung cancer mutation prediction, Yong et al. [27] on loss of rare cell populations in scRNA‐seq integration, Venet et al. [1] on the apparent significance of random gene signatures, and Kabir and Wong [24] on DeepFam’s collapse in the twilight zone of low‐similarity sequences. When treated as pedagogical resources rather than isolated critiques, such studies transform methodological failure into structured learning opportunities for the community. Normalizing this form of analysis shifts the field away from a “publish and forget” culture toward a self‐correcting ecosystem in which artifacts, shortcuts, and evaluation failures are systematically exposed and studied. Strengthening this culture of transparency and biological grounding is essential if AI is to mature into a reliable and scientifically meaningful partner in biological discovery.

## 10 CONCLUDING REMARKS

With the rapid and accelerating adoption of AI in computational biology, the field stands at a critical juncture. Current practices may either establish AI as a transformative tool that advances the discovery of biological knowledge and its translation into medical applications, or allow it to devolve into a facade of superficially impressive but scientifically fragile results. Persistent inflation of claims risks eroding public trust in computational biology, whereas unchecked methodological weaknesses may become normalized, leading to a gradual erosion of scientific standards.

Institutional efforts to enforce rigor are already emerging in response to these concerns. For example, *Bioinformatics* now requires validation on independent datasets to support claims of generalizability. Clinical AI venues such as *Radiology: Artificial Intelligence* and journals within the JAMA Network similarly mandate evaluation on data from independent institutions to mitigate shortcut learning and ensure robustness across populations. In addition, the *Journal of Bioinformatics and Computational Biology* explicitly requires evidence of substantive methodological contribution, transparent and representative data curation, independent evaluation sets, and deployment‐aware assessment practices [19].

These expectations must extend beyond specialized biomedical venues to include broader AI and computing‐focused communities, including IEEE and related conferences and journals. Although such venues often emphasize architectural innovation, the validity of any technical contribution ultimately depends on the rigor of its evaluation. An apparent architectural improvement demonstrated only on convenient or “AI‐ready” datasets, where hidden confounders, data leakage, or the doppelgänger problem are not adequately controlled, may reflect a methodological illusion rather than a genuine advance. For the two communities to coexist productively, technical venues must also recognize that model performance is meaningful only when it is robust to biological complexity and consistent with deployment realities.

To support this shift, we have distilled the core requirements of rigorous AI research into the concise set of five VALID principles (see Supporting Information S1). This framework is intended as a practical tool for researchers, reviewers, and editors to anchor evaluation in biological relevance, methodological rigor, and deployment realism. Although the transition to these higher standards may be gradual, meaningful change is achievable if leading researchers collectively reinforce them through practice. As Feynman [36] famously cautioned, “The first principle is that you must not fool yourself—and you are the easiest person to fool.” Adopting a higher standard of evidence ensures that AI is used as a tool for genuine scientific discovery, rather than allowing insufficient rigor to define the trajectory of the field. Importantly, these standards do not depend on large‐scale computational resources; they primarily require intellectual rigor, domain expertise, and a commitment to scientific integrity.

## AUTHOR CONTRIBUTIONS

**Tsung Fei Khang**: Investigation; writing—original draft; writing—review and editing. **Joshua W. K. Ho**: Investigation; writing—review and editing. **Lin Hou**: Investigation; writing—review and editing. **Jiangning Song**: Investigation; writing—review and editing. **Limsoon Wong**: Conceptualization; investigation; project administration; writing—original draft; writing—review and editing.

## CONFLICT OF INTEREST STATEMENT

The authors declare no conflicts of interest.

## Supporting information

Supporting Information S1

## Data Availability

Data sharing is not applicable to this article as no datasets were generated or analyzed during the current study.
