# Supplementary material for: The evaluation crisis in artificial intelligence‐driven computational biology: Pitfalls and solutions
Source: Quant Biol. 2026 Sep 23;14(4):e70058. doi: 10.1002/qub2.70058 (PMC13642056; doi:10.1002/qub2.70058)
Supplement: Supplementary file 1 [file QUB2-14-e70058-s001.docx]

## The VALID Guiding Principles for Rigorous AI in Biology

*To move beyond superficial performance and ensure that AI models deliver genuine scientific insight, the following principles serve as a framework for authors, reviewers and editors. Rather than serving as a passive checklist, these principles offer a set of reflective prompts designed to ensure that the core requirements of rigorous science—validity, robustness, and utility—are upheld throughout the research lifecycle.*

**Principle 1: Veracious Biological Grounding and Novelty**

- **Reflective Prompt:** Does the model facilitate the discovery of genuinely novel biological insights, or does it merely recapitulate established knowledge through tuned parameters?
- **The Goal:** To move beyond black-box predictive exercises toward models that provide mechanistic leads or experimentally testable hypotheses.

**Principle 2: Artifact Control and Methodological Rigor**

- **Reflective Prompt:** What specific tests were performed to rule out “shortcut learning,” such as exploiting batch effects and dataset leakage? Have synthetic datasets with known ground truths been used to verify authentic signal capture?
- **The Goal:** To ensure that the model learns intrinsic biological signals rather than dataset artifacts.

**Principle 3: Legitimate Generalization**

- **Reflective Prompt:** Does the evaluation design, such as the use of time-splits, lab-splits, or low-similarity sequence partitioning, and independent datasets, faithfully reflect the distribution shifts, hard cases, and unexpected novel cases the model will encounter in real-world deployment?
- **The Goal:** To avoid convenience splits that inflate performance and instead focus on hard cases and out-of-distribution robustness.

**Principle 4: Inspectable Preprocessing**

- **Reflective Prompt:** Are all preprocessing choices (e.g., normalization, imputation, and batch correction) documented transparently, and has their impact on the biological signal been quantified?
- **The Goal:** To enable reproducibility and verify that success is not driven by unintended artifacts in the hidden preprocessing steps.

**Principle 5: Deployment-Aware Performance Metrics**

- **Reflective Prompt:** Are the reported performance metrics evaluated at operationally relevant thresholds, and do they rigorously account for prevalence, uncertainty, and deployment expectations in the intended environment?
- **The Goal:** To shift from raw metric gains to empirical evidence that satisfies real-world domain constraints and practical reliability.
